# Imaging of parotid anomalies in infants and children

**DOI:** 10.1186/s13244-022-01166-y

**Published:** 2022-02-24

**Authors:** François Chalard, Anne-Laure Hermann, Monique Elmaleh-Bergès, Hubert Ducou le Pointe

**Affiliations:** 1 Department of Pediatric Radiology, Hôpital Armand Trousseau, 26, Avenue du Dr. Arnold Netter, 75012 Paris, France; 2 Pediatric Radiology, Hôpital Robert Debré, Paris, France

**Keywords:** Parotid gland, Imaging, Children

## Abstract

A wide spectrum of disorders involves the parotid glands, in infancy and childhood. Acute or chronic inflammatory/infectious diseases are predominant. The first branchial cleft anomalies are congenital lesions that typically manifest during childhood. Tumor lesions are more likely to be benign, with infantile hemangioma the most common in infancy and pleomorphic adenoma the most frequent in childhood. Malignant tumors are uncommon, with mucoepidermoid carcinoma the least rare. Infiltrative parotid diseases are rare and have some pediatric clinical specificities. These common and uncommon disorders of parotid glands during childhood and their imaging characteristics are reviewed.

## Key points

- The causes of parotid gland anomalies in children differ from those in adults.
- The main parotid disorders are inflammatory and infectious diseases.
- Parotid tumors are more likely to be benign (infantile hemangioma in infancy and pleomorphic adenoma in childhood).
- The most frequent malignant tumor is mucoepidermoid carcinoma.
- Congenital cystic anomalies must be recognized, to avoid repeated infections

## Background

### Imaging tools

With high-frequency transducers, sonography provides high-resolution images of superficial tissues such as salivary glands [1]. Being readily achievable, non-invasive (notably non-ionizing) and a low-cost modality, ultrasonography is the first-line technique to explore parotid anomalies in children [2]. It differentiates rapidly parotid from non-parotid lesions and indicates if the lesion is cystic, vascular or solid. When further exploration is needed, CT or MRI may be considered. In acute situation, when an inflammatory or infectious disorder is suspected, when a lithiasis is sought or when looking for a bone involvement, CT may be performed. However, when a parotid tumor is suspected and/or deep structures must be explored, MRI is the best imaging modality [3, 4]. MRI provides high contrast resolution images, non-invasive sialography, diffusion-weighted sequences and dynamic contrast-enhanced sequences (DCE-MRI) [5]. Morphological MRI images assess the size, the location and the content of a neoplasm. Multi-parametric MR imaging helps in distinguishing benign from malignant tumors, based on studies using a combination of some of the following parameters: ADC (apparent diffusion coefficient), TIC (time intensity curve), Ve (extracellular volume ratio), permeability MRI, TBF (tumor blood flow) [6–10]. Moreover, the feasibility of intraparotid facial nerve MR tractography in estimating the presence of a contact between the facial nerve and a tumor has recently been shown, in adults [11]. However, the precise location of the facial in the parotid gland is not routinely assessed. Digital subtraction sialography is no more used.

### Embryology reminder

The parotid gland is the first major salivary gland to develop, between 4 and 8 weeks’ of gestation. It arises from an epithelial bud of the floor of the primitive mouth, which invaginates into the underlying mesenchyme, proliferates and forms lobules of secretory parenchymal tissue. A groove develops in this bud, canalizes and branches, leading to Stensen’s canal and its canaliculi. During that period, the parotid gland and the facial nerve have an entangled growth, practically embracing each other [12].

Finally, the parotid gland has a triangular shape and is located at the angle of the mandible, below the external auditory canal. It lies posteriorly to the masseter muscle and the ramus of the mandible, lateral to the parapharyngeal and carotid spaces and anteriorly to the mastoid and the sternocleidomastoid muscle [2]. It contains the retro mandibular vein, the external carotid artery and the facial nerve, the latter dividing the gland into superficial and deep lobes and being not commonly seen on sonography.

**Fig. 1:**
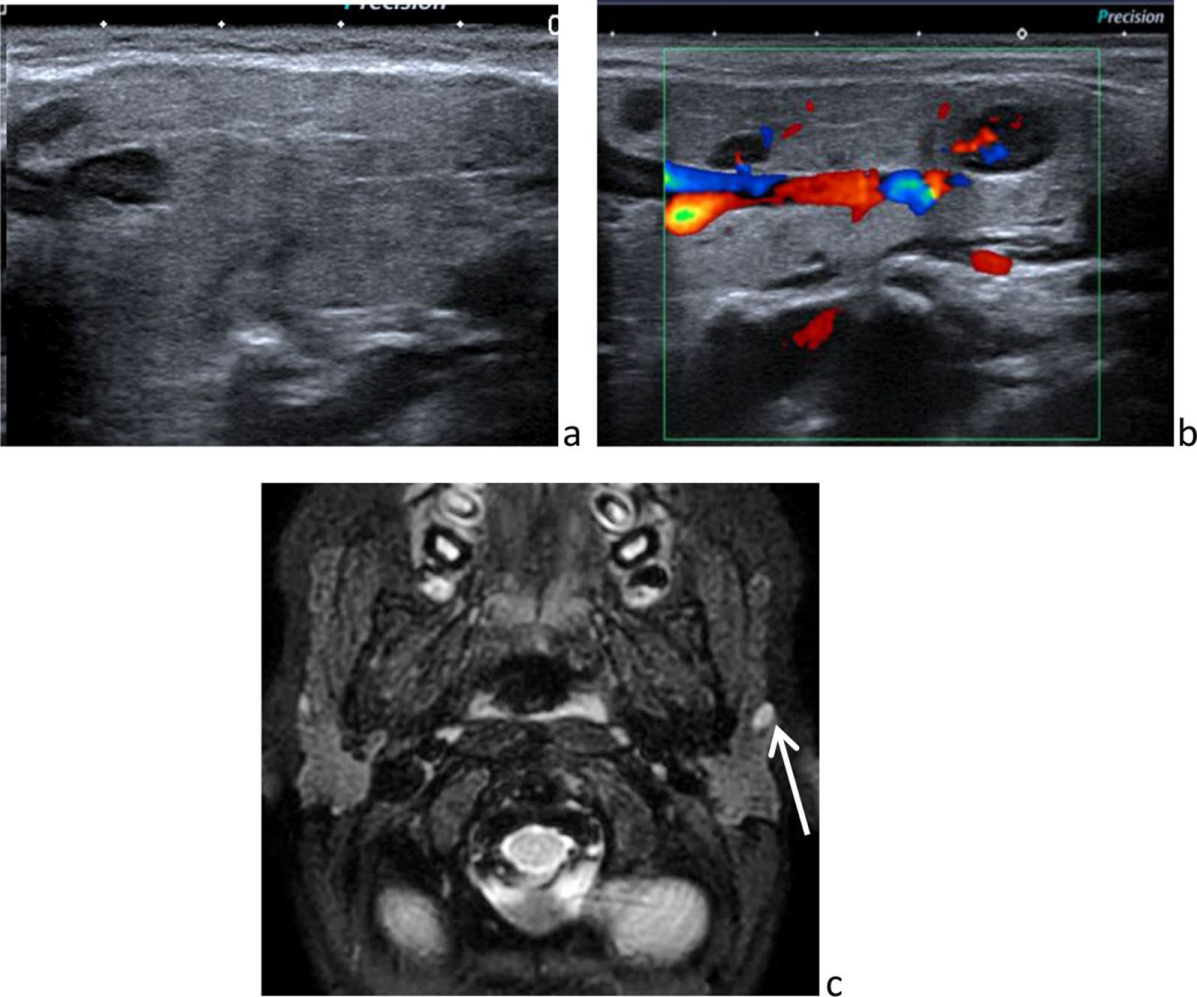
Intraparotid normal lymph nodes on sonography (**a**), color-Doppler sonography (**b**) and axial fat sat *T*_2_-weighted image (**c**)

Unlike the submandibular gland, the parotid gland also contains lymph nodes and lymphatic channels, because of its late encapsulation during development (Fig. 1) [13]. This explains the occurrence of parotid lymphoid/lymphatic disorders.

**Fig. 2:**
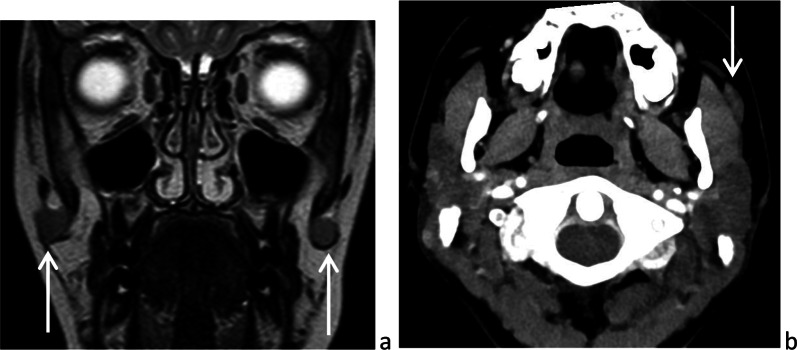
Accessory parotid glands on frontal *T*_2_-weighted image (**a**) and axial CT after intravenous injection of contrast medium (**b**)

The facial process of the parotid gland is the anterior extension of the gland, to which it is connected. The accessory parotid gland is a nodule of normal tissue separated from the main parotid gland, located on the masseter muscle or more anteriorly and connected to Stensen’s duct at that level (Fig. 2) [14]. Rarely, lesions may occur in the accessory parotid gland, such as the main gland. These lesions, which can mimic other cheek lesions, should be appropriately recognized as parotid lesions on imaging.

## Parotid gland anomalies

### Parotitis

Parotitis is defined as inflammation of the parotid gland, which may have various causes, including infectious (viral or bacterial), autoimmune, lithiasic or idiopathic causes [15, 16]. Infectious sialadenitis is more common in parotid than other salivary glands. In contrast, sialolithiasis is much rarer in parotid than in submandibular glands and has no specific imaging appearance.

#### Acute viral parotitis

**Fig. 3:**
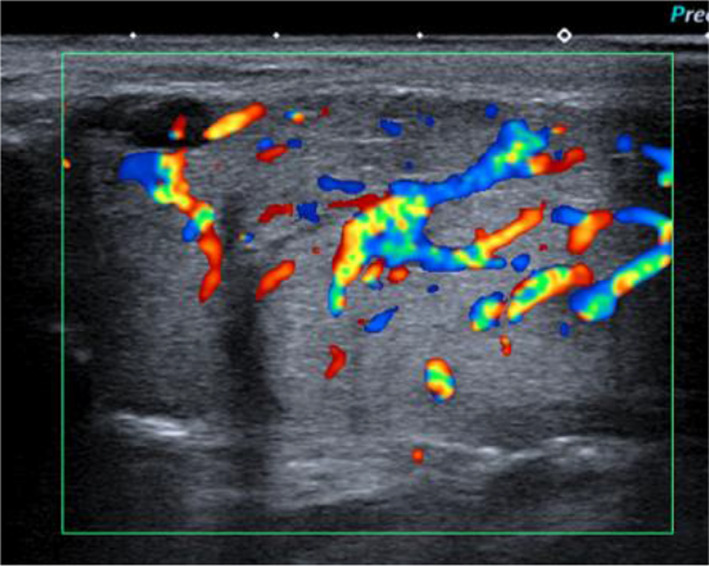
Acute viral right parotitis. Enlarged, hypervascularized parotid on ultrasonography

Acute viral parotitis is more common than bacterial parotitis and is frequently bilateral (90% of cases) but not always synchronously. The main viruses are paramyxovirus (mumps, whose incidence has greatly reduced due to vaccination), Epstein-Barr virus, cytomegalovirus, parainfluenza and HIV. Clinical signs are painful swelling of the parotid gland, fever and inflammation of the orifice of Stensen’s duct. Occasionally, the submandibular and sublingual glands are also affected [17]. Imaging is not needed for the diagnosis. When performed, ultrasonography shows a glandular enlargement with foci of salivary secretions, lymph nodes and increased vascularization (Fig. 3). Spontaneous regression in a few days is the rule.

#### The special case of human immunodeficiency virus (HIV)

Note that HIV children prevalence rate is low, in western countries. Indeed, if the estimated 1.72 million children under 15 years of age are living with HIV, 89 per cent live in sub-Saharan Africa [18].

**Fig. 4:**
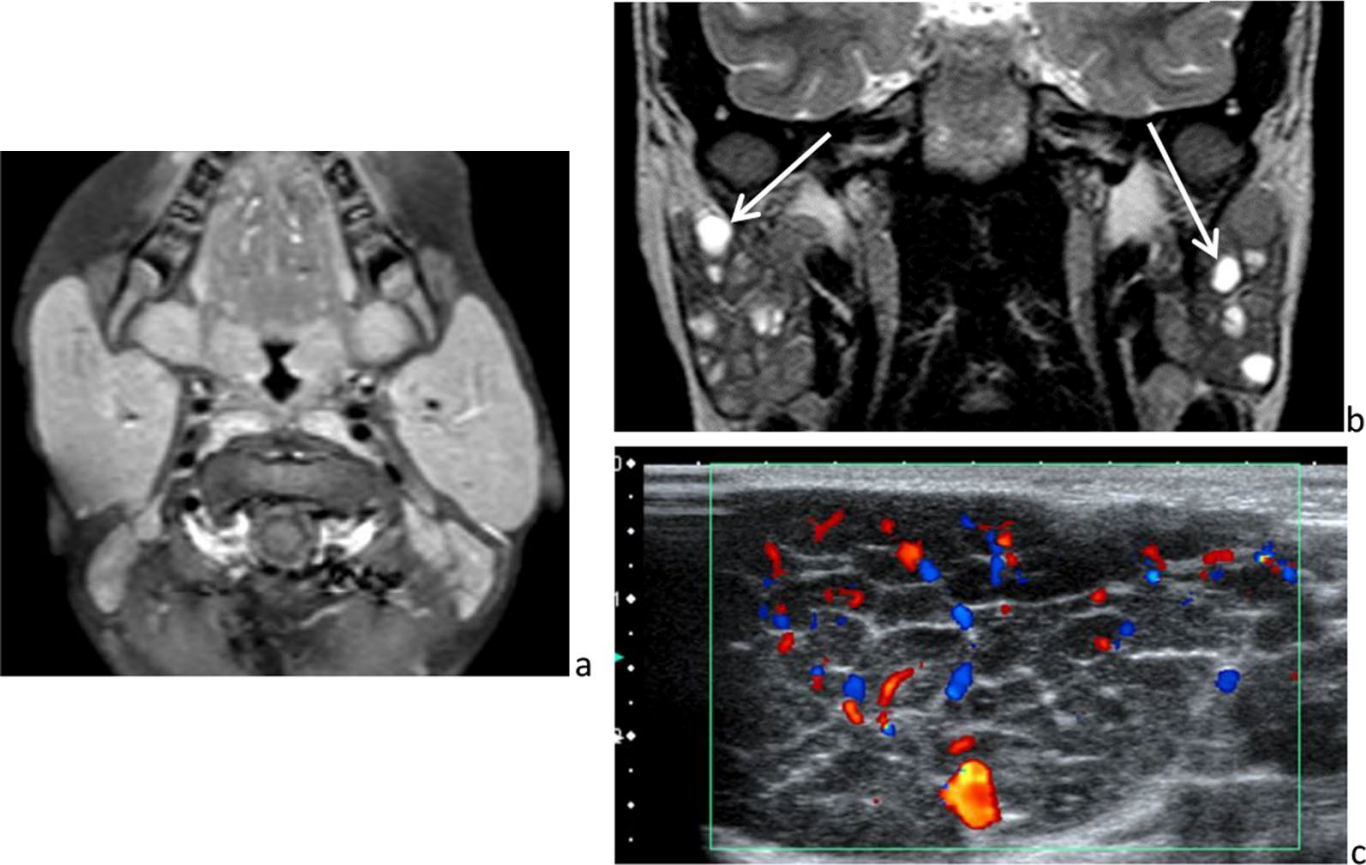
HIV-related disorders in 3 different patients. Diffuse homogeneous parotid enlargement on axial *T*_1_-weighted image, after intravenous administration of gadolinium chelate (**a**), lympho-epithelial cysts on frontal *T*_2_-weighted image (**b**) and benign lympho-epithelial lesions, hypoechoic on ultrasonography (**c**)

In addition to causing acute parotitis, HIV infection may cause persistent generalized lymphadenopathy (including intraparotid lymph nodes), lympho-epithelial cysts and benign lympho-epithelial lesions [19]. These lesions occur early in the course of the retroviral infection and consist of lymphocytic infiltration, intraductal epithelial proliferation and atrophy of salivary acini. Sonography and MRI reveal a diffuse glandular enlargement, multiple thin-wall cysts of variable size and/or multiple solid masses and cervical lymphadenopathies (Fig. 4). These lesions may also be present in disorders such as Sjogren’s syndrome and sarcoidosis. With HIV infection, there is also increased risk of malignant lymphoma [20].

#### Acute bacterial parotitis

**Fig. 5:**
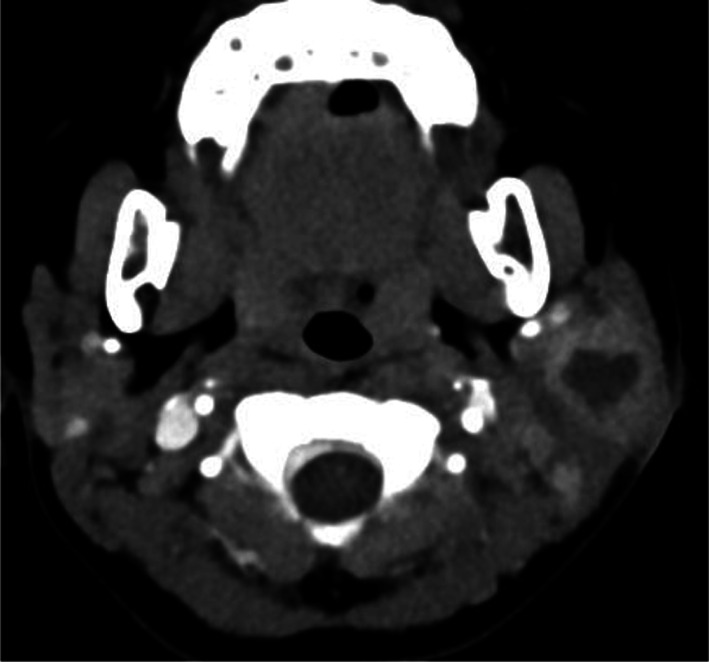
Left parotid gland abscess on CT, after intravenous injection of contrast medium

Acute bacterial parotitis may be due to direct inoculation of the ductal epithelium or ascending oral cavity infection. The most common pathogens associated with acute bacterial parotitis are *Staphylococcus aureus* and *Streptococcus spp.*, and less commonly, anaerobic pathogens [15]. Unlike viral parotitis, acute bacterial parotitis presents as by painful swelling, fever, erythema of the overlying skin and sometimes trismus. Pus may be manually expressed from Stensen’s duct. Urgent ultrasonography and/or computed tomography (CT) may be required to search for local complications, suppurated parotitis and parotid abscess (Fig. 5), which are seen as fluid collection(s) within an enlarged hypervascular gland. Rarely is lithiasis found in Stensen’s duct. Intravenous antibiotic therapy is the cornerstone of treatment [21].

#### Cat scratch disease

**Fig. 6:**
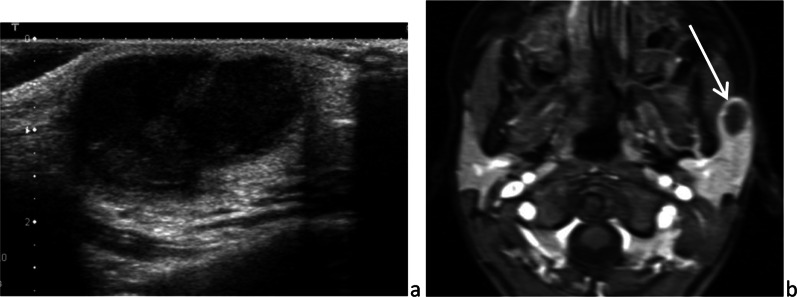
Cat scratch disease. Left, intraparotid necrotic lymphadenitis: ultrasonography (**a**); axial T_1_-weighted image, after intravenous administration of gadolinium chelate (**b**)

Cat scratch disease is an infection caused by *Bartonella henselae*, a Gram-negative bacteria transmitted by a cat scratch or bite. It is typically a chronic lymphadenopathy involving the upper limb or the head and neck, including the parotid gland. Imaging workup shows necrotic lymphadenitis: hypoechoic lymph node with peripheral hypervascularization on ultrasonography [22], and a hypodense lymph node with rim enhancement and surrounding soft tissue edematous swelling on CT (Fig. 6) [23]. These imaging features may mimic malignant disorders (metastasis, sarcoma or hemopathy) or other infectious, pyogenic or mycobacterial conditions. The diagnosis should be raised in young children exposed to cat(s) and confirmed by serology or PCR of a lymph node biopsy. In most cases, the clinical evolution is spontaneously favorable, and antibiotics are reserved for severe or systemic disease.

#### Tuberculosis and atypical mycobacterial infection

Tuberculosis of salivary glands is most frequently seen after systemic dissemination of pulmonary infection. Its most frequent location is the parotid gland. Clinically, it may present as acute bacterial parotitis with few local inflammatory signs or have an indolent course and mimic a parotid tumor. On imaging, it has the aspect of acute parotitis, which tends to form an abscess or necrotic nodes, later calcified [20, 24].

**Fig. 7:**
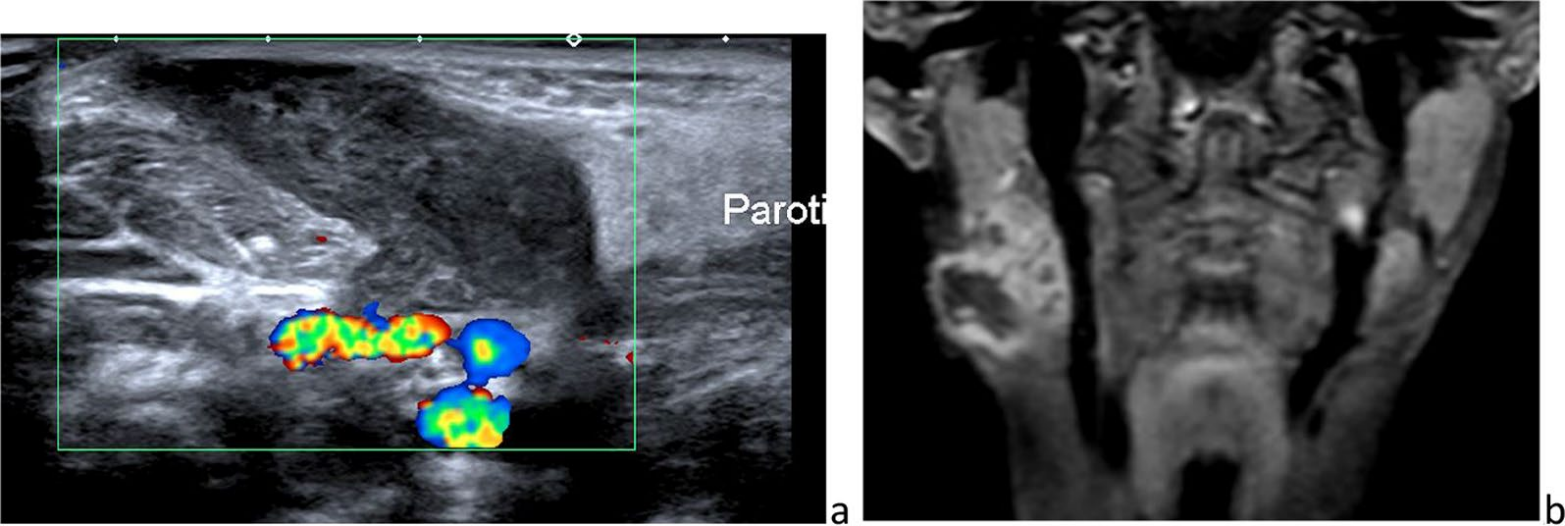
Mycobacterial infection. Necrotic lymphadenitis located immediately below the parotid gland and complicated by a skin fistula: sonography (**a**) and frontal T_1_-weighted image, after intravenous administration of gadolinium chelate (**b**)

Atypical mycobacterial infection is caused by *Mycobacterium avium* or *Mycobacterium intracellulare,* occurs in immunocompetent children and is thought to be an extension of cervical adenitis*.* It presents as a slow-growing, painless mass that is difficult to distinguish from tuberculosis and can lead to a skin fistula, highly suggestive of the diagnosis (Fig. 7) [20, 25].

#### Juvenile recurrent parotitis (JRP)

**Fig. 8:**
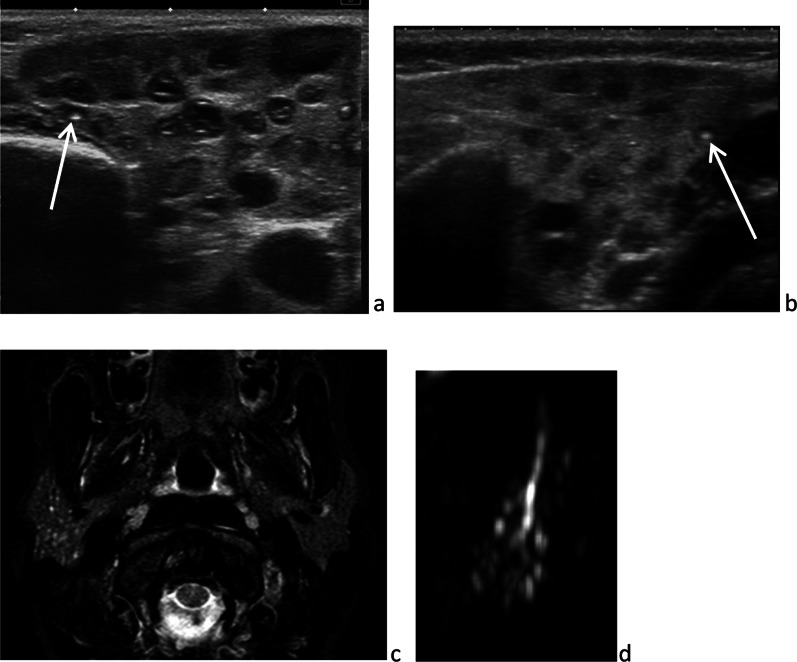
Juvenile recurrent parotitis. Multiple foci of salivary secretions scattered in a normal sized gland, with some central calcification [white arrow] and without Stensen’s duct dilatation, on ultrasonography (**a** and **b**), axial fat sat *T*_2_-weighted image (**c**) and MR-sialography (**d**)

JRP is the most common inflammatory salivary gland disease in childhood and adolescence, with many assumed and possibly interwoven causes: genetic, immune, infection, dehydration, allergy, ductal abnormalities and ductal obstruction [12]. JRP begins in early childhood and resolves in adolescence. It may be unilateral or bilateral. Patients present repeated episodes of painful swelling of the gland, sometimes complicated by a bacterial infection. Between two episodes, the volume of the gland returns to normal or, at least, decreases. At the end of the disease course, the parotid gland may be hard and mimic a tumor on clinical examination. Ultrasonography may be sufficient for diagnosis, showing multiple hypoechoic foci of salivary secretions, scattered in the gland and inconstantly containing central calcification (Fig. 8a, b). Vascularization of the gland is a normal appearance, on Doppler color imaging [26]. According to the stage of the disease, early or late, the parotid gland is either enlarged or atrophic [23]. When a second imaging modality is needed, MR sialography can confirm the diagnosis. MRI reveals a non-dilated Stensen’s duct and multiple small “cystic” round areas of high signal on T_2_-weighted images (mimicking a bunch of grapes), in an enlarged gland (Fig. 8c, d) [27]. Treatment is not consensual: antibiotics, analgesics, sialendoscopy with unclogging [24].

### Congenital anomalies

Congenital anomalies of the parotid are mainly venolymphatic malformations and branchial cleft cysts. They may occur in isolation or as part of a syndrome.

The new classification of the International Society for the Study of Vascular Anomalies distinguishes vasoproliferative lesions (e.g., infantile and congenital hemangiomas, tumors with increased endothelial cell turnover) and vascular malformations (with normal endothelial cell turnover). The latter may be high-flow malformations (arteriovenous malformation or fistula) or low-flow malformations (capillary, venous, lymphatic or mixed) [28].

#### Lymphatic malformation or lymphangioma

**Fig. 9:**
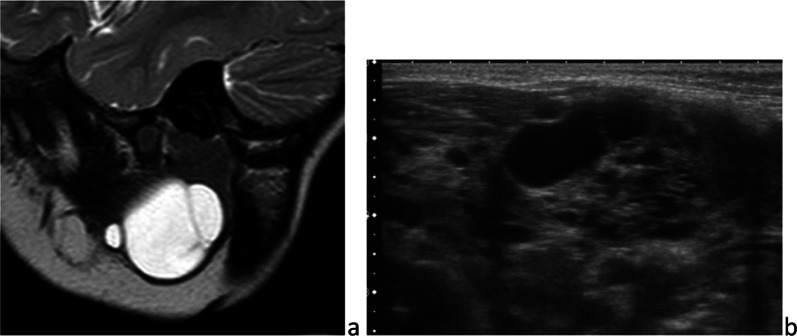
Lymphatic malformations. Macrocystic lymphangioma on parasagittal *T*_2_-weighted image (**a**) and micro- and macrocystic lymphangioma on ultrasonography (**b**)

These malformations of the lymphatic system can be macrocystic, microcystic or mixed. They are composed of dilated lymphatic channels surrounded by lymphoid tissue. Ubiquitous lymphangiomas are likely found in the neck, including the parotid gland. Some are diagnosed prenatally, especially if they are macrocystic; 50% are detected at birth and 90% manifest before age 2 years. They are most often sporadic and may occur in syndromes such as Turner and Noonan and trisomies 13, 18 and 21 [20]. In the absence of complications, lymphangioma are soft painless masses. On imaging, whatever the techniques, lymphangiomas appear as multiple thin-walled cystic structures of variable sizes, whose echogenicity and MR signals are very close to those of water (Fig. 9). Thin vascularized septa classically separate the cysts. Because lymphatic malformations are likely trans-spatial masses, they can spread outside the parotid gland to deep cervical spaces and/- or subcutaneous fat, insinuating between normal structures. If they become large, they may significantly compress adjacent structures, resulting in respiratory difficulty or dysphagia [29]. In case of infection or post-traumatic hemorrhage, they suddenly increase in size, become hard and tender or painful and are associated with local inflammatory signs. On imaging, the cysts that have grown in size present a fluid–fluid level and/or increased echogenicity and signal, on *T*_1_-weighted images [30]. Spontaneous involution is possible but rare. Treatment depends on the size of the cysts, its mainstay being sclerotherapy and surgical excision.

#### Venous malformations (VM)

**Fig. 10:**
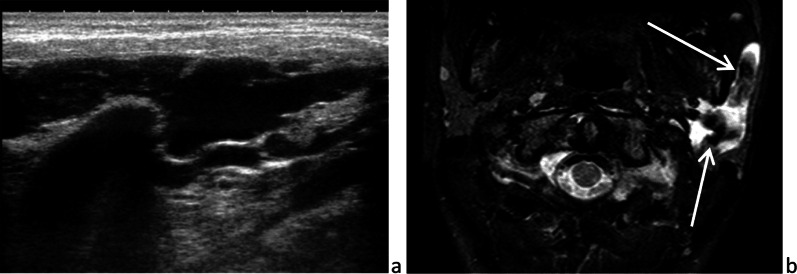
Venous malformation. Tortuous veins containing phleboliths, on sonography (**a**) and *T*_2_*-weighted image (**b**)

VMs are low-flow vascular malformations classified into two types: cavitary and dysplastic [31]. In the absence of thrombosis, they are painless compressible masses. The covering skin can be bluish if the mass extends to the subcutaneous region. On sonography, cavitary VMs appear as infiltrative compressible lesions, whereas dysplastic VMs, rarer, consist of multiple tortuous veins (Fig. 10a). Phleboliths, round calcifications in a vein, may be present and have to be searched for, as a key sign of the diagnosis of VMs. Pulse-Doppler ultrasonography reveals a monophasic low flow, whose detection is increased by the Valsalva maneuver and/or compression. For cavitary VMs, the flow can be very slow and therefore difficult to detect. MRI helps in defining the extension of the malformation and its drainage. Fat suppression *T*_2_-weighted sequences clearly delineate the lesion: highly intense as compared with surrounding structures, notably the fat spaces. Gradient echo *T*_2_-weighted images reveal phleboliths, nodular elements of very low signal (Fig. 10b). *T*_1_-weighted images show hemorrhage and thrombosis as an area of hypersignal. Sequences performed after intravenous injection of gadolinium provide information relative to perfusion and drainage of VM [31]. A multidisciplinary approach is desirable, to decide whether treatment is necessary and what modality must be chosen: sclerotherapy, drugs (e.g., Sirolimus), surgery or laser.

#### First branchial cleft anomalies

**Fig. 11:**
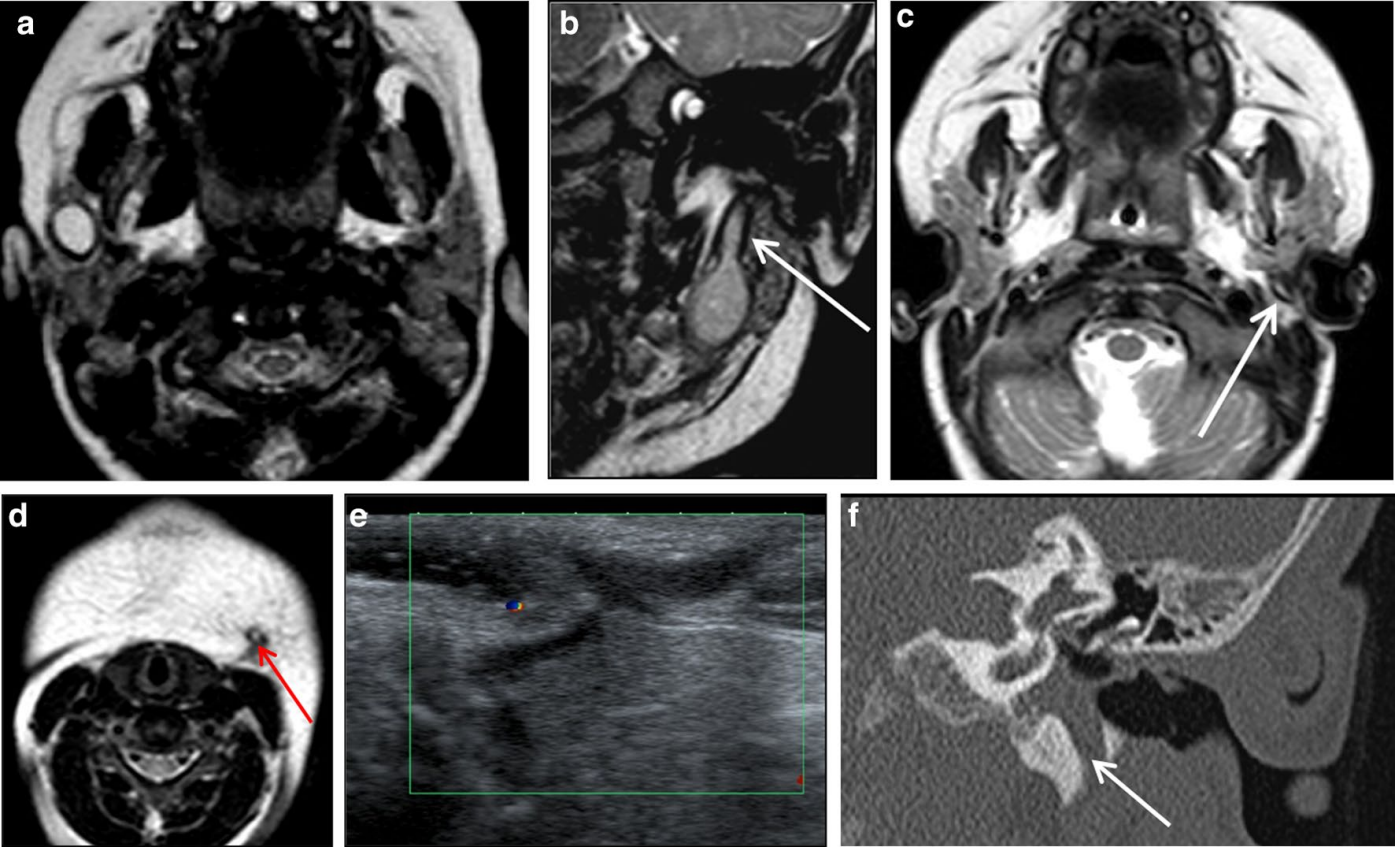
First branchial-cleft cyst and fistulas. Right isolated intraparotid cyst on axial *T*_2_-weighted image (**a**). Type II anomaly, with an intraparotid cyst and 2 fistulas, the upper fistula running from the cyst to the external auditory canal [white arrow] and the lower running from the cyst to the skin, in Pochet’s triangle [red arrow] (**b**, **c** and **d**). Isolated fistula running from the parotid area to the skin (**e**). Congenital defect of the floor of the left external auditory canal (tympanal bone) on frontal and axial CT images (**f** and **g**)

The branchial apparatus is a transient structure formed from the 4–7 week’s of gestation. It consists of paired branchial arches separated by pharyngeal pouches, branchial grooves and branchial membranes and will give rise to definitive cervical structures. The failure of involution of the pouches, grooves and/or membranes leads to malformations consisting of cysts (68% of cases), sinuses (16%) and fistulas (16%) [32]. Congenital first branchial-cleft anomalies are classified into two groups. Type I anomalies are cysts or a sinus opening medial, inferior or posterior to pinna and conchal cartilage. The cyst is located in the parotid gland or the immediate periparotid area, and the sinus runs parallel to the external auditory canal. Type II anomalies are more common and considered a duplication of the external auditory canal. They consist of a sinus running from the floor of the external auditory canal through parotid gland to the neck, the skin opening of the sinus localized above the hyoid bone, in Pochet’s triangle [33]. First branchial-cleft anomalies may be asymptomatic, in the absence of infection. When infection occurs, the patient presents otorrhea and/or a tender, inflamed parotid mass and a biological inflammatory syndrome. On imaging, the cyst appears as a round collection located in the parotid gland or close to it, with a thin-smooth wall (Fig. 11a). In case of infection, the wall of the cyst becomes thicker and more irregular, like an abscess, and surrounded by edema. Tracts (sinuses and fistulas) are easier to assess with MRI or CT than sonography. They appear as lines or pipes running from the submandibular region to the external auditory canal and/or skin, at the upper level of the anterior cervical triangle (Fig. 11b–e). In addition, CT may show associated bone anomalies, such as defect in the floor of the external auditory canal (i.e., the tympanal bone) (Fig. 11f, g). With infection, these tracts have irregular-enhancing borders. Appropriate treatment requires complete surgical excision of the malformation in addition to medical treatment of infection. If the patient receives only medical treatment, recurrence is inevitable [34]. Therefore, in case of “parotid abscess,” particularly if recurrent, a first branchial-cleft cyst should be considered.

#### Aplasia

**Fig. 12:**
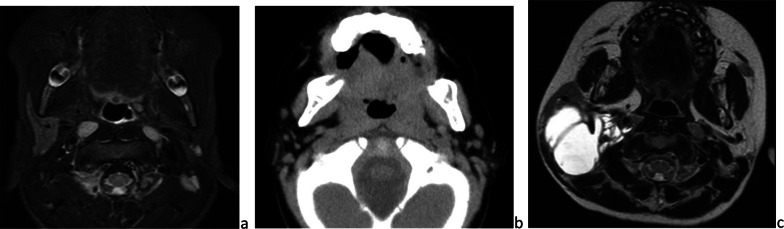
Parotid gland aplasia. Unilateral, isolated aplasia on axial fat sat *T*_2_-weighted image (**a**). Bilateral aplasia on axial CT image, in Treacher-Collins syndrome (**b**). Right parotid gland aplasia associated with contralateral macrocystic lymphangioma, on axial *T*_2_-weighted image (**c**)

Resulting from a defect in embryonal development, aplasia or agenesis can affect any salivary gland. Commonly unilateral, parotid-gland aplasia is likely associated with other congenital disorders such as hypoplasia of other salivary glands or the lacrimal glands or with cranio-oral malformations: cleft lip/palate, lacrimo-auriculo-dento-digital syndrome, hemifacial microsomia or congenital anomalies of the first and second branchial arches such as Treacher-Collins syndrome (Fig. 12a–c) [35]. Bilateral parotid gland aplasia leads to chronic xerostomia and its complications: dental caries, oropharyngeal infections (candidiasis), halitosis, periodontal disease and chewing and swallowing disorders. Management is based on dietary and oral hygiene advice (teeth brushing, hydration) and caries treatment [36].

### Tumors

Salivary gland tumors are uncommon (1% of all pediatric tumors) and are mostly benign in children., more than 90% occurring in the parotid gland [24]. In childhood, mesenchymal tumors are more common than epithelial tumors. The commonest parotid gland tumor is the hemangioma, with a mesenchymal origin, and is benign. Hemangioma is particularly frequent in infants younger than 1 year, representing 90% of all parotid tumors, at this age [13]. The second most frequent parotid tumor and the most frequent epithelial-benign tumor is pleomorphic adenoma (mixed tumor) [37]. It classically occurs in late childhood or adolescence, where it is the most frequent neoplasm. The most frequent malignant tumor is mucoepidermoid carcinoma, with an epithelial origin [38]. Mucoepidermoid carcinoma represents 50% of all malignant parotid tumors and generally occurs between 5 and 15 years [39]. Imaging may guide the diagnosis and may be suggestive (typically with infantile hemangioma), but the precise diagnosis of a parotid tumor generally requires histopathology analysis.

### Benign tumor

Infantile hemangiomas and pleomorphic adenomas are the two most common benign tumors of the parotid gland.

#### Infantile hemangioma

**Fig. 13:**
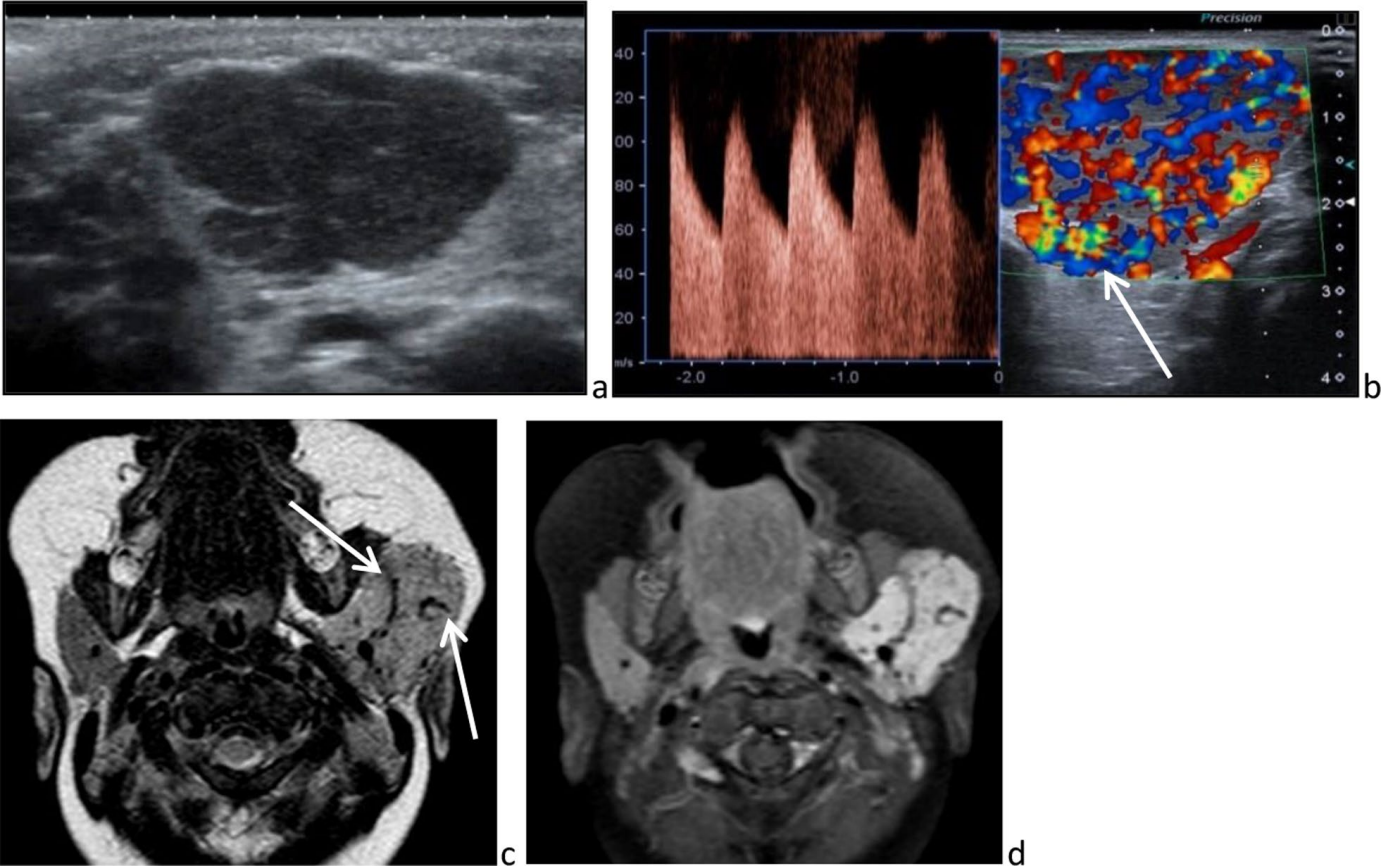
Infantile hemangioma. Lobulated, hypoechoic and hypervascular mass (**a**) containing high velocity and low resistance arteries (**b**), on ultrasonography. Diffuse enlargement of the left gland by strongly enhanced mass containing tortuous vessels responsible for flow void images [white arrows], on axial *T*_2_-weighted image (**c**) and T_1_-weighted image, after intravenous injection of gadolinium chelate (**d**)

According to the new classification of the International Society for the Study of Vascular Anomalies, a hemangioma is a vasoproliferative lesion. It is both the most common soft tissue tumor in infancy and the most prevalent benign salivary gland tumor in children. Histologically, it is an unencapsulated mass of packed, thin-walled capillaries and endothelial cells, positive for GLUT-1 [25]. It manifests one or two months after birth, with a female predominance, as a soft mass of the parotid area, associated or not with an adjacent cutaneous infantile hemangioma. Then, it follows a classical evolution: rapid growth until a peak at age one to two years (proliferative phase) then gradual spontaneous regression, generally complete by adolescence (involutive phase) [40]. On ultrasonography, hemangiomas are lobulated, hyperechoic and/or hypoechoic and hypervascular masses, which may enlarge the entire parotid gland (Fig. 13a). Both arteries and veins are visible on color Doppler ultrasonography and spectral Doppler ultrasonography shows high-velocity and low-resistance arteries (Fig. 13b). Rarely, a direct arteriovenous shunt is seen, which should not be interpreted as an arteriovenous malformation [31]. On MRI, hemangiomas are well-limited lobulated masses, without any surrounding edema. They have an intermediate signal on T_1_-weighted images and a hypersignal on T_2_-weighted images, contain flow void corresponding to fast-flow vessels and are strongly enhanced (Fig. 13c, d). In the involutive phase, hemangiomas reduce in size, have a reduced number of vessels and become heterogeneous. This heterogeneity is due to fibrosis or fat involution, well recognized on MRI (fibrosis has a hyposignal on *T*_1_ and *T*_2_-weighted image and fat has a hypersignal on *T*_1_ and *T*_2_ sequence) and sonography (hyperechogenicity). Because of the spontaneous involution, treatment is recommended only with complications such as ulceration, infection, bleeding and severe growth leading to compression and/or deformity. Beta blockers are the first-line treatment for infantile hemangiomas; embolization and laser therapy are second-line treatments [41]. Of note, hemangiomas, especially segmental ones, are part of the PHACES syndrome (posterior fossa malformation, carotid and cerebral artery anomalies, cardiac defects, aortic coarctation, eye anomalies and sternal defects), in 2% to 3% of cases [42].

#### Pleomorphic adenoma

**Fig. 14:**
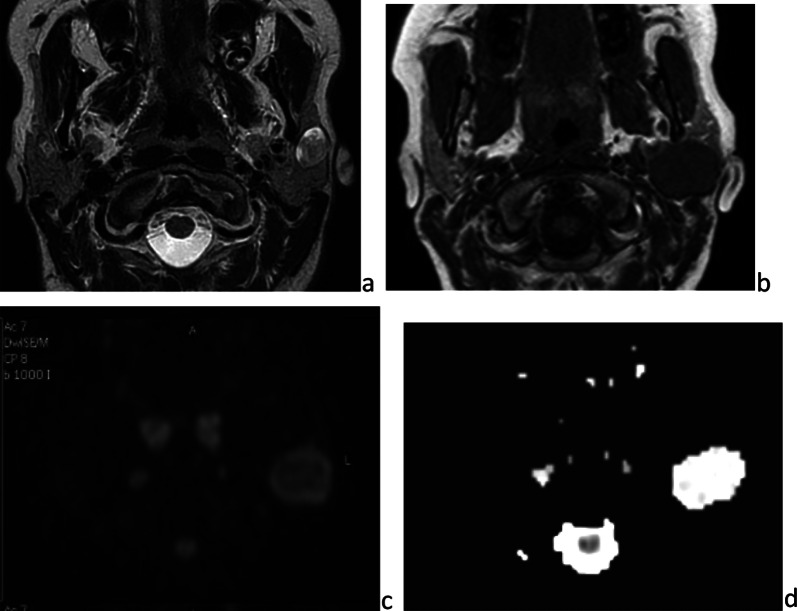
Pleomorphic adenoma or mixed tumor. Axial T_2_-weighted image showing the hypointense capsule well delineating the tumor (**a**). At a different slice level, axial *T*_1_-weighted image (**b**)_,_ diffusion weighted image (**c**) and apparent diffusion coefficient map (**d**) showing a homogeneous tumor with a very high apparent diffusion coefficient

Also named benign mixed tumor, of epithelial origin, pleomorphic adenoma is the second most frequent benign tumor of the parotid gland. It occurs in late childhood or adolescence, the median age of onset being 15 years [24]. It typically presents as a painless, slow-growing firm or hard mass, which progressively enlarges. On imaging, it appears as a well-defined, encapsulated tissue mass (Fig. 14a). Ultrasonography reveals a hypo or isoechoic mass versus normal parotid gland that may contain some small calcifications. MRI often shows the hypointense capsule on *T*_2_-weighted images. The MR signal depends on the size of the tumor. If the tumor is small, its signal is homogeneously hypointense on *T*_1_-weighted image and hyperintense on *T*_2_-weighted images. For larger tumors, the MR signal is heterogeneous because of necrosis, hemorrhage and/or cysts. Tumor enhancement is mild, more or less homogeneous, also depending on the tumor size. Generally, the apparent diffusion coefficient (ADC) is high in pleomorphic adenoma, as is it classically in benign tumors [23, 25] (Fig. 14b–d). Besides, the pleomorphic adenoma has a persistent or flat TIC pattern on DCE-MRI [7] and a low TBF [8]. Treatment consists of facial-nerve-sparing parotidectomy and not just enucleation, to minimize the risk for recurrence [43].

#### Neurofibroma

**Fig. 15:**
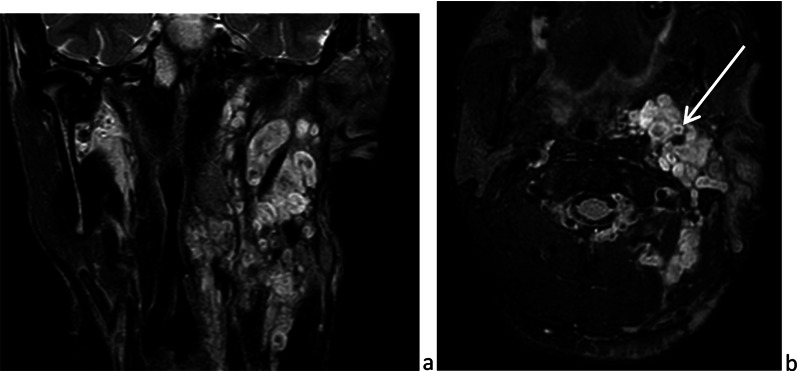
Neurofibroma, in a child with neurofibromatosis type 1. Frontal and axial *T*_2_-weighted images (**a** and **b**). Tumor infiltrating the left parotid gland and surrounding spaces. The NF is globally hyperintense (myxoid tissue), with multiple central hyposignals (collagen fibers): “target sign” [white arrow]

Neurofibroma is a benign nerve-sheath tumor infiltrating between the nerve fascicles. In the parotid gland, it arises from the facial nerve trunk or its branches. When it is solitary, the neurofibroma is generally isolated [23]. When it is plexiform or multiple, it may be associated with neurofibromatosis type 1 (von Recklinghausen disease). On sonography, it is approximately round and heterogeneous, its centre hyperechoic and its periphery hypoechoic. On CT, its central part is hypodense and cystic-like, whereas its periphery is enhanced moderately. On MRI, neurofibroma is iso or hypointense on *T*_1_-weighted images and hyperintense on *T*_2_-weighted image. Sometimes it presents the “target sign” on *T*_2_-weighted images, the central hypointense dot corresponding to collagen and the hyperintense peripheral ring corresponding to myxoid tissue, typical of plexiform neurofibroma, whatever its location (Fig. 15). Conversely, the tumor may contain heterogeneous areas of signal intensity, for difficulty distinguishing it from pleomorphic adenoma [24, 25]. Treatment, mainly consisting of surgery, is reserved for some painful or rapidly growing lesions, which may cause cosmetic damage.

A non-exhaustive list of other benign tumors of the parotid gland includes the following neoplasms: Warthin tumor (adenolymphoma or papillary lymphomatous cystadenoma), much rarer than in adults, often located in the tail of the parotid and possibly bilateral [25], as well as angiolipoma, oncocytoma, hamartoma, sialolipoma [44], dermoid cyst and adenoma. An exceptional case of unicentric Castleman disease, benign lymphoproliferative disorder of unknown origin, has even been reported [45].

### Malignancies

Pediatric salivary gland malignancies are rare, particularly before age 10 years [46], representing 5% of all salivary gland malignancies [47]. Such lesions occur more frequently in parotid glands than in submandibular, sublingual or minor salivary glands.

Parotid tumors are more frequently malignant in children (up to 50%) than adults [48]. In childhood, the main group of these malignant lesions consists of epithelial tumors, mucoepidermoid carcinoma being the most frequent neoplasm, followed by acinic cell carcinoma, adenoid-cystic carcinoma and adenocarcinoma, together representing > 80% of carcinomas [16, 43, 49]. Next are mesenchymal tumors, essentially represented by rhabdomyosarcomas, malignant hemopathies (lymphomas and leukemias), metastasis and the exceptional sialoblastoma. Clinically, these lesions are more often symptomatic than benign lesions (pain, facial nerve palsy, adenomegaly).

**Fig. 16:**
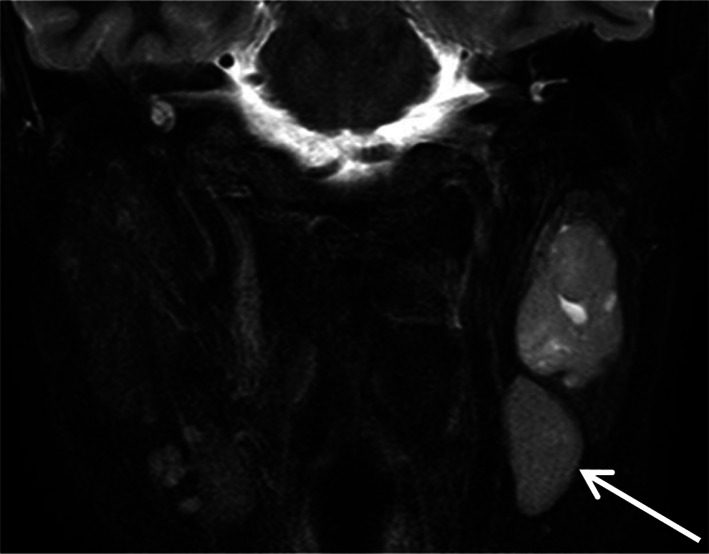
Acinic cell carcinoma. Satellite lymphadenomegaly on frontal fat sat *T*_2_-weighted image

On imaging, some criteria are suggestive of malignancy despite some overlap with benign lesions: ill-defined limits, irregular areas of hyposignals on *T*_2_-weighted images, restricted diffusion [50], TIC with a signal growing rapidly in the first phase and decreasing gradually in the second phase (type C, according to Yabuuchi et al.) [6, 9], high tumor blood flow (TBF) [8] and lymphadenomegaly (Fig. 16).

#### Epithelial carcinomas

**Fig. 17:**
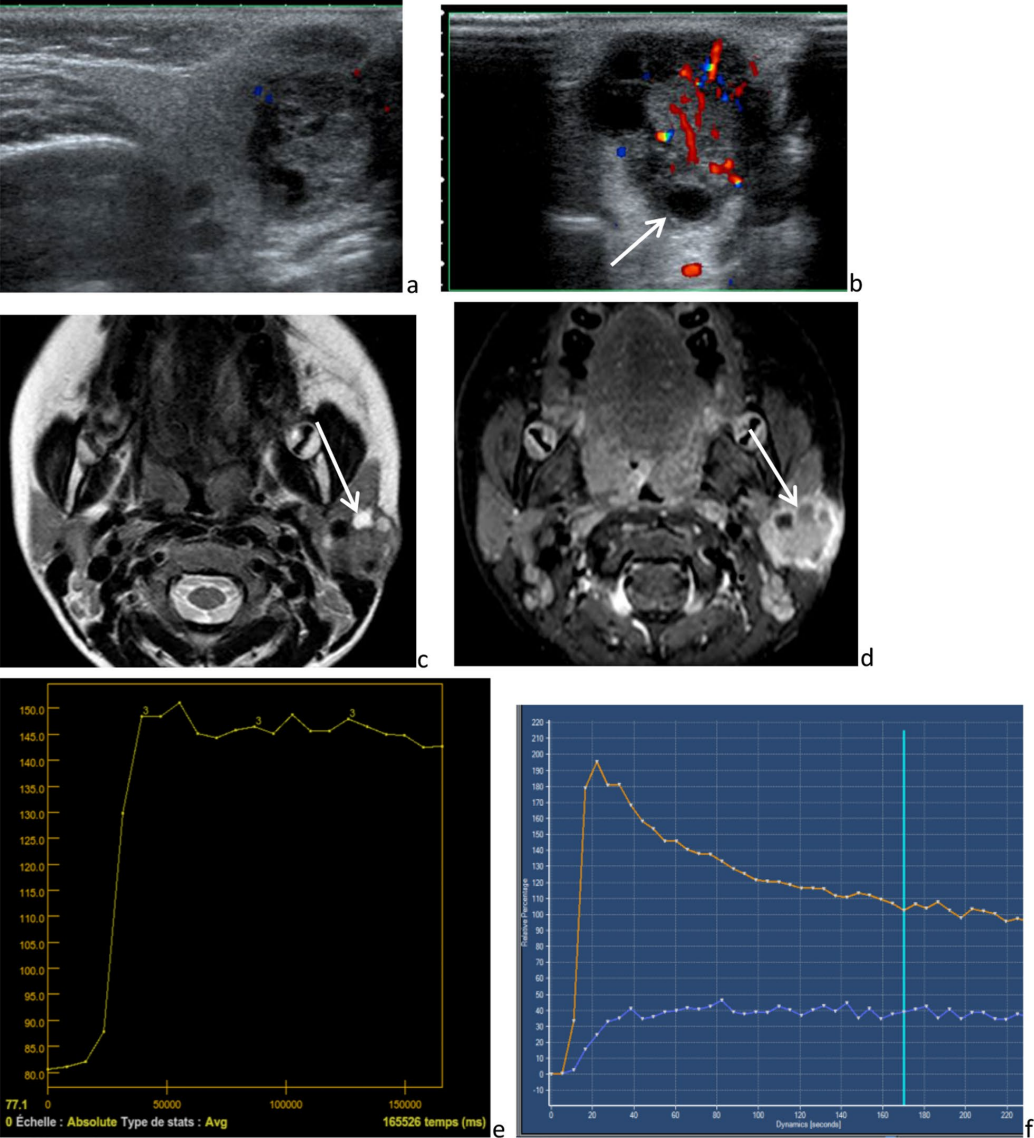
Intermediate-grade mucoepidermoid carcinoma: poorly delineated, heterogeneous tumor containing small necrotic areas [white arrow] and strongly enhanced, on ultrasonography (**a** and **b**), axial *T*_2_-weighted image (**c**) and *T*_1_-weighted image, after intravenous injection of gadolinium chelate (**d**). Time intensity curve with a signal growing rapidly in the first phase and a low wash out (< 30%): type C or plateau pattern (according to Yabuuchi), suggestive of malignancy (**e**). Conversely, time intensity curve of type B (orange line), with a high wash out (> 30%), in a case of parotid benign lymphadenopathy (**f**)

Mucoepidermoid carcinoma is the main malignant tumor in childhood and is generally a low-grade lesion. The presentation is an asymptomatic, palpable swelling of the parotid area, with or without regional lymphadenopathy. On imaging, the aspect of the tumor is variable (Fig. 17). According to Kashiwagi et al. [51], the imaging aspect seems to depend on histologic characteristics. Low-grade tumors feature a hyperintense area on *T*_2_-weighted images, related to abundant mucin secretant cells, and sometimes ill-defined margins, because of peritumoral inflammatory changes. High-grade tumors feature ill-defined margins and intermediate to low signals on *T*_2_-weighted images, reflecting invasive tumor growth and high cellularity, respectively. The ADC is low in mucoepidermoid carcinoma, as in malignant tumors as compared with benign lesions [52]. On DCE-MRI, the TIC curve type is suggestive of malignancy. Lymphadenopathies are inconstantly found associated with high-grade tumor.

Surgery is the primary treatment of parotid-gland carcinomas, whose overall survival is better in children than adults, owing to the better localized and differentiated nature [53]. Adjuvant radiotherapy may be indicated with unfavorable prognosis, whereas the role of chemotherapy is limited, reserved for some inoperable or metastatic lesions [54]. Muco epidermoid carcinoma has relatively good prognosis, even if it has a tendency for recurrence [55].

#### Rhabdomyosarcoma

**Fig. 18:**
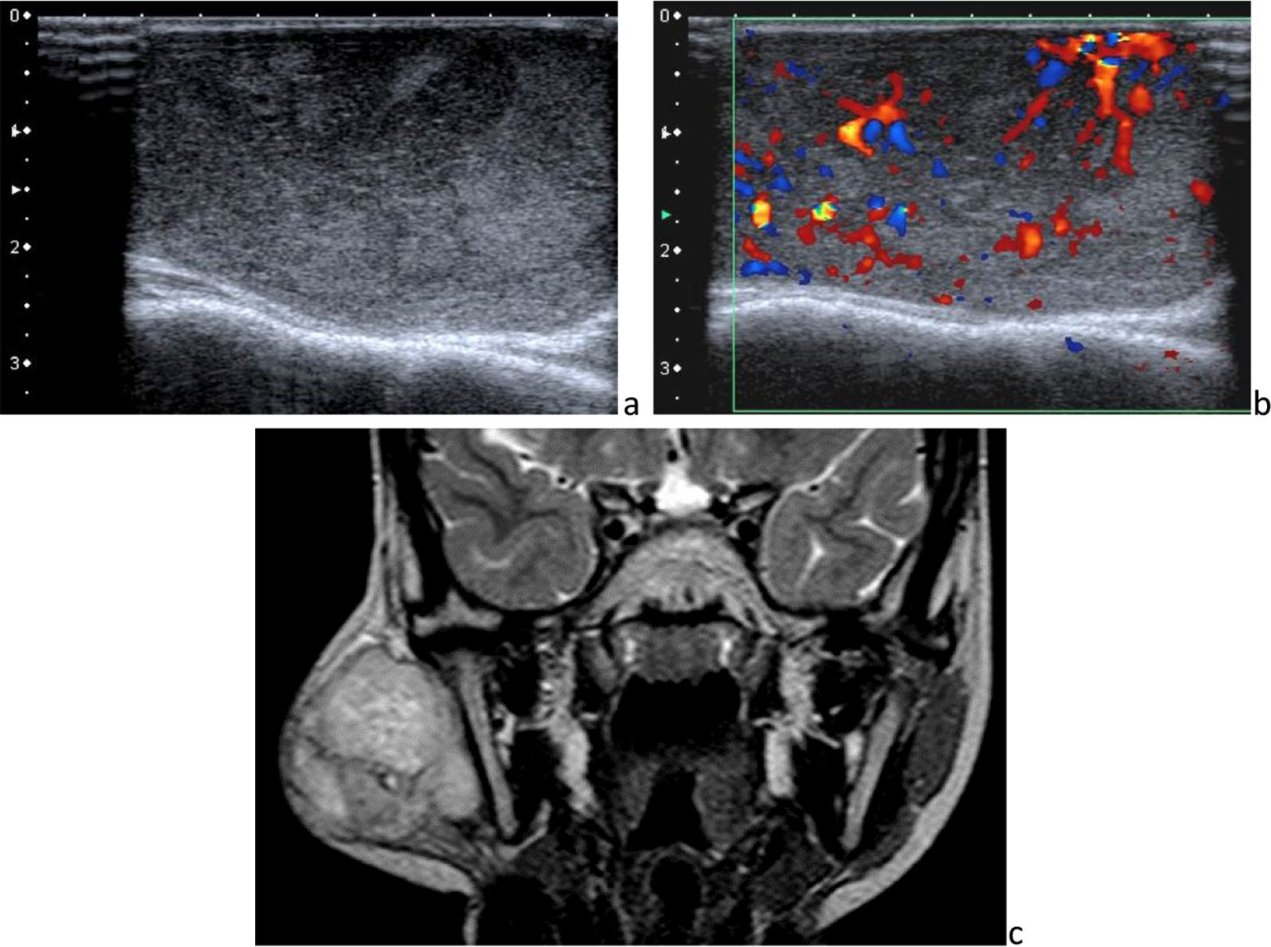
Embryonic rhabdomyosarcoma. Heterogeneous structure and vascularization on B-mode sonography and color Doppler (**a** and **b**), large at the time of diagnosis on frontal T_2_-weighted image (**c**)

Rhabdomyosarcomas are the most frequent pediatric soft tissue malignant tumors, encountered in 40% of cases in the head and neck, mainly the orbits, nasopharynx, middle ear, nasal cavity and paranasal sinuses [20]. Embryonal rhabdomyosarcoma is the most common form, representing 60% of cases [30]. Two peak ages of presentation are 2–5 years and 15–19 years. Parotid gland involvement classically occurs by direct extension. Rhabdomyosarcomas are poorly defined masses whose spontaneous density or signal is variable, depending on the presence of necrosis (Fig. 18). They generally have low ADC, enhance diffusely and mildly and have a TIC curve suggestive of malignancy, on DCE-MRI. Locally aggressive, they may cause adjacent bony destruction, which has to be searched for, as well as regional lymphadenopathy. Rhabdomyosarcomas have reserved prognosis because patients often present advanced disease, which tends to recur. Treatment combines chemotherapy and surgery and sometimes radiotherapy.

#### Sialoblastoma

Also named congenital tumor embryoma or congenital basal adenoma, sialoblastoma is a rare salivary gland tumor diagnosed in the fetus, newborn or infant. Initially classified as a benign tumor, it is now considered a malignant tumor. Histologically, it shows an arrested state of salivary maturation [56]. Locally aggressive, it may cause skin deformities, superficial hemorrhage, ulceration and necrosis. It has no specific imaging and its treatment is surgery, with adjuvant chemotherapy if complete excision is not possible.

#### Lymphoma and leukemia

Lymphomas are uncommon in the parotid gland. Primary lymphomas, classified as MALTomas, arise from mucosal lymphoid tissue, and secondary lymphomas represent a true parenchymal pathology of the salivary gland. On imaging, lymphomas diffusely and homogeneously infiltrate the gland they enlarge or lead to nodular lesions [25]. With diffuse infiltration, the parotid gland shows increased flow on Doppler ultrasonography and is rather homogeneous on CT and MRI, before and after intravenous injection of contrast medium. An important feature is a decreased ADC, due to the hypercellularity of lymphoma. Bilateral parotid-gland involvement is frequent.

**Fig. 19:**
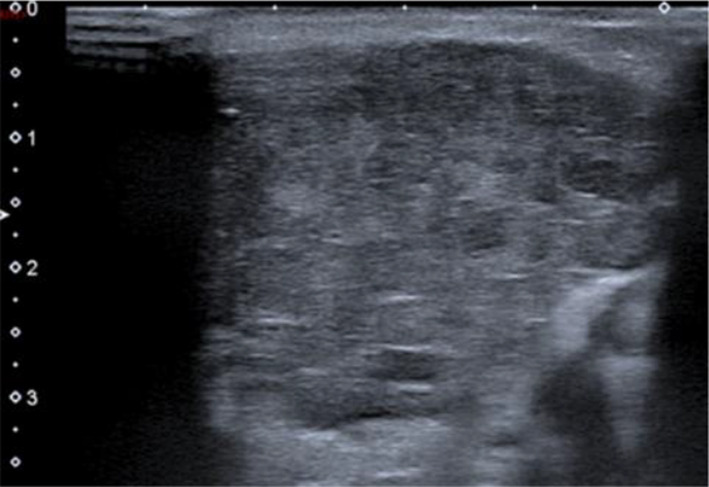
Acute leukemia. Heterogeneous infiltration leading to bilateral parotid gland enlargement, on ultrasonography

Leukemic infiltration is also rare [57, 58] and cannot be distinguished from other infiltrative diseases by only imaging (Fig. 19). Diagnosis is based on peripheral blood and bone-marrow smear analysis. Here again, bilateral lesions are common.

#### Metastasis

Even if quite exceptional in childhood, squamous cell carcinoma, periauricular melanoma and thyroid carcinoma may spread in the parotid lymph nodes [59]. The diagnosis is suggested by a careful examination of the face and neck and is based on the pathologic examination.

### Other disorders

#### Sialadenosis or sialosis

**Fig. 20:**
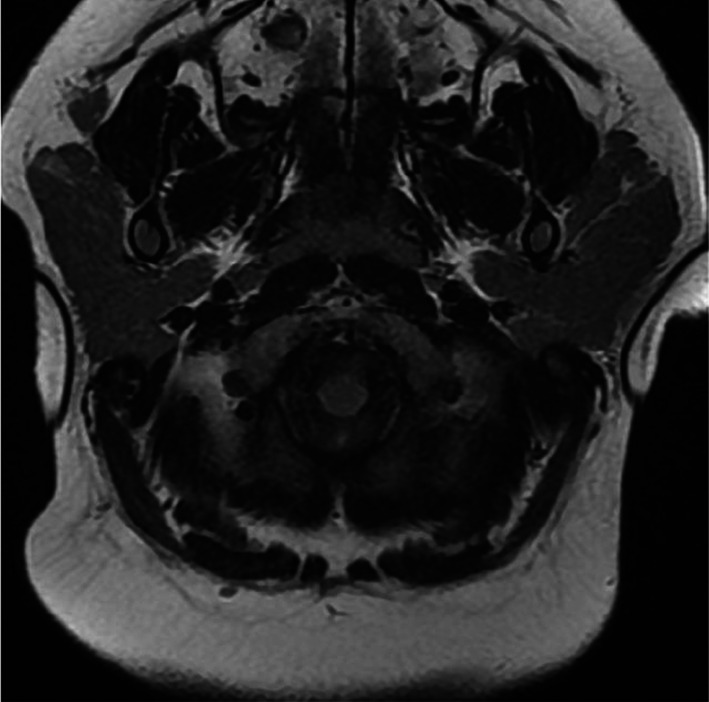
Sialadenosis in an obese teenager. Diffuse enlargement of both parotid glands whose signal is normal and abundant subcutaneous fat, on axial *T*_2_-weighted image

Sialadenosis or sialosis is a non-inflammatory, non-tumor bilateral swelling of the major salivary glands (mainly the parotid glands), which can be painful or asymptomatic. In adults, sialadenosis is most commonly due to alcoholism, endocrine disorders such as diabetes, nutritional disorders or drugs. During late childhood and adolescence, the main causes are eating disorders such as bulimia and anorexia. Sonography shows enlarged, homogeneous parotids, whose diffuse hyperechogenicity may limit the visibility of their deep lobe [60]. MRI also shows diffuse and homogeneous hypertrophy of both parotid glands (Fig. 20). The conditions feature neither focal anomaly nor hypervascularization. Major differential diagnoses of chronic, bilateral swelling of parotid glands are sarcoidosis, Sjogren’s syndrome, malignant hemopathies, Epstein-Barr virus and human immunodeficiency virus. Cases of Langerhans cell histiocytosis have been reported, but are exceptional [61].

#### Sarcoidosis (or Besnier-Boeck-Schaumann disease)

**Fig. 21:**
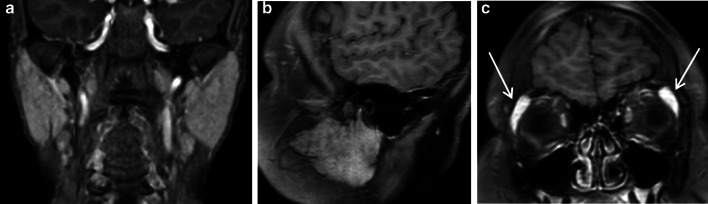
Sarcoidosis. Bilateral infiltration and strong, homogeneous enhancement of parotid glands, on frontal and parasagittal fat sat *T*_1_-weighted image (**a** and **b**) and lacrimal glands, on frontal fat sat *T*_1_-weighted images (**c**), after intravenous administration of gadolinium chelate

Sarcoidosis is a systemic disorder, a non-caseating granulomatous disease of unknown origin. It has a wide spectrum of manifestations, with lungs and lymph nodes usually affected [62]. Sarcoidosis is rare in children. Children have the same clinical and radiological signs as adults. Infants may present skin, eye or joint symptoms, without any lung involvement [20]. Parotids are affected in about 30% of cases, and in 6% of cases the disease is limited to these glands. Generally, enlargement of both glands is painless. On imaging, glands are homogeneously enlarged, have intense signals on *T*_2_-weighted images and are hypervascularized (Fig. 21). Cervical lymph nodes are likely enlarged. Sometimes the parotids contain multiple non-cavitating masses [25]. Heerfordt syndrome combines bilateral parotiditis, fever, uveitis and facial nerve palsy. Mikulicz syndrome associates bilateral infiltration of parotid and lacrimal glands, which occur in other infiltrative diseases, such as Sjogren’s syndrome.

#### Sjogren’s syndrome (SS)

SS is a chronic autoimmune disorder that affects mainly middle-aged women. In children, two forms are distinguished: primary and secondary (associated with another autoimmune disease, such as juvenile rheumatoid arthritis). SS consists of lymphocytic infiltration followed by parenchymal destruction, which primarily involves the salivary and lacrimal glands, causing a dry syndrome (keratoconjunctivitis sicca and xerostomia). Extra-glandular manifestations (arthritis, vasculitis, pulmonary fibrosis, nephropathy and neuropathy) occur in a few patients [63]. SS, particularly the primary form, is even rarer in childhood than in adulthood, and symptoms of dry eye and xerostomia develop later during the disease [64]. Patients commonly present recurrent episodes of acute, tender parotid swelling and progressive glandular enlargement. Sonography reveals glandular enlargement and heterogeneity due to hyperechoic foci of mucoid impactions and hypoechoic ductal dilatations [43]. Later on, foci of lymphocytic aggregates, calcifications and atrophy may be seen. MRI, including MR sialography, is an excellent technique for diagnosis and staging. Indeed, MRI shows a punctate, globular, cavitary or destructive appearance in the parotid gland, associated with the phase of the disease [65]. Primary SS carries a long-term risk for malignant degeneration into lymphoma.

#### Pneumoparotid and pneumoparotitis

**Fig. 22:**
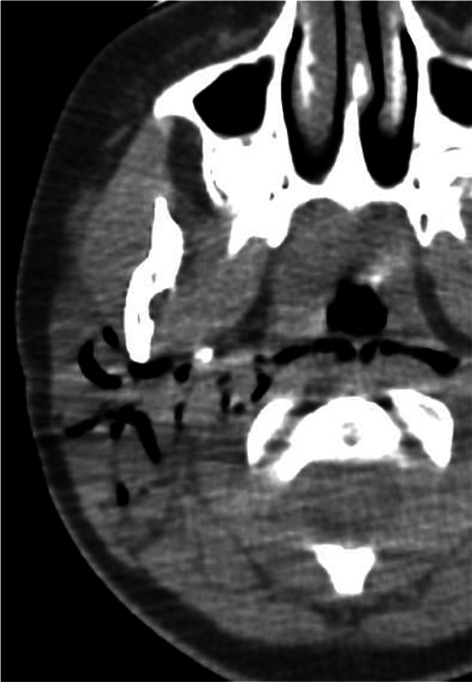
Right pneumoparotid complicated with deep cervical emphysema, of unknown etiology, on axial CT image. *Courtesy Dr. M. Mabille (CHI Créteil, France)*

Pneumoparotid is defined as the presence of air within the parotid gland. This condition is due to increased intraoral pressure, leading to retrograde insufflation of air in the Stensen’s duct and the parotid canaliculi. Revealed by an inconstantly painful swelling of the cheek, it is bilateral in 50% of cases. There is sign of local inflammation. CT is the gold-standard technique to diagnose a pneumoparotid (Fig. 22). In adults, it has been described in glass blowers, wind instrumentalists, divers, after dental instrumentation (braces), in coughing attack or in psychiatric patients [66, 67]. In children and teenagers, it has been reported in case of psychosocial issues, the cause of the pneumoparotid being a self-induced disorder, as in psychiatric adult patients. Pneumoparotid is often complicated with subcutaneous emphysema and, less frequently, with pneumomediastinum and inflammation/infection. The latter complication is named pneumoparotitis. Treatment is adjusted to the cause and the consequence of the disorder: antibiotics, psychological counseling, dental braces adjustment, etc.

## Pseudoparotid lesion

**Fig. 23:**
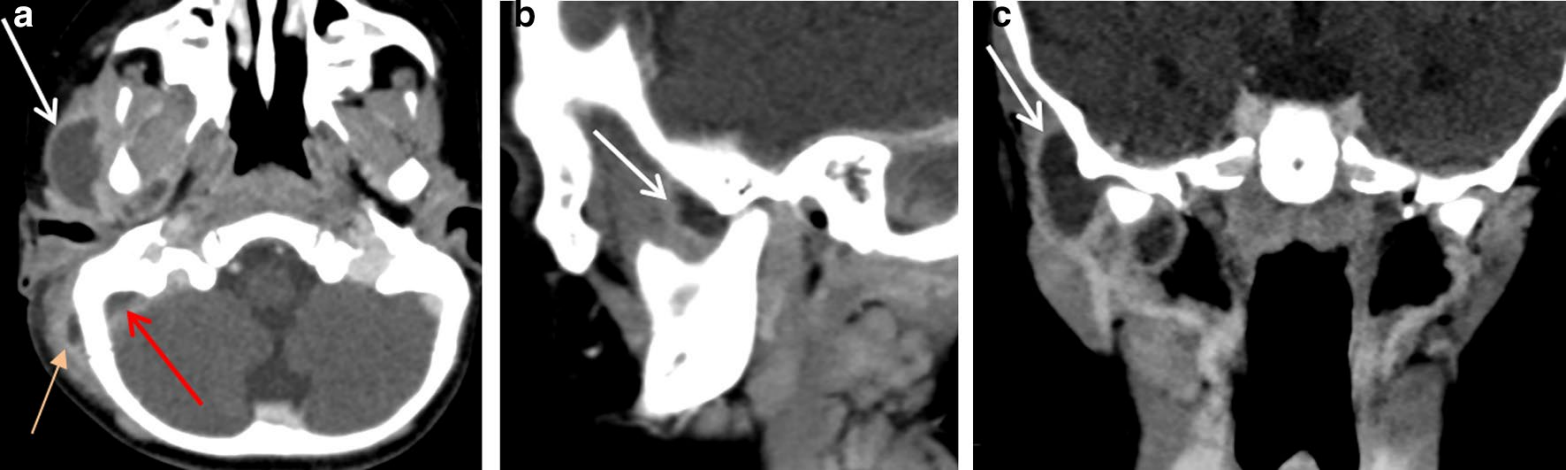
Fusobacterium necrophorum infection. Right otomastoiditis complicated by subperiosteal abscess [orange arrow], sigmoid sinus thrombosis [red arrow] and temporomandibular arthritis [white arrow] leading to cheek swelling, on axial, parasagittal and frontal CT images, after intravenous injection of contrast medium (**a**, **b** and **c**)

**Fig. 24:**
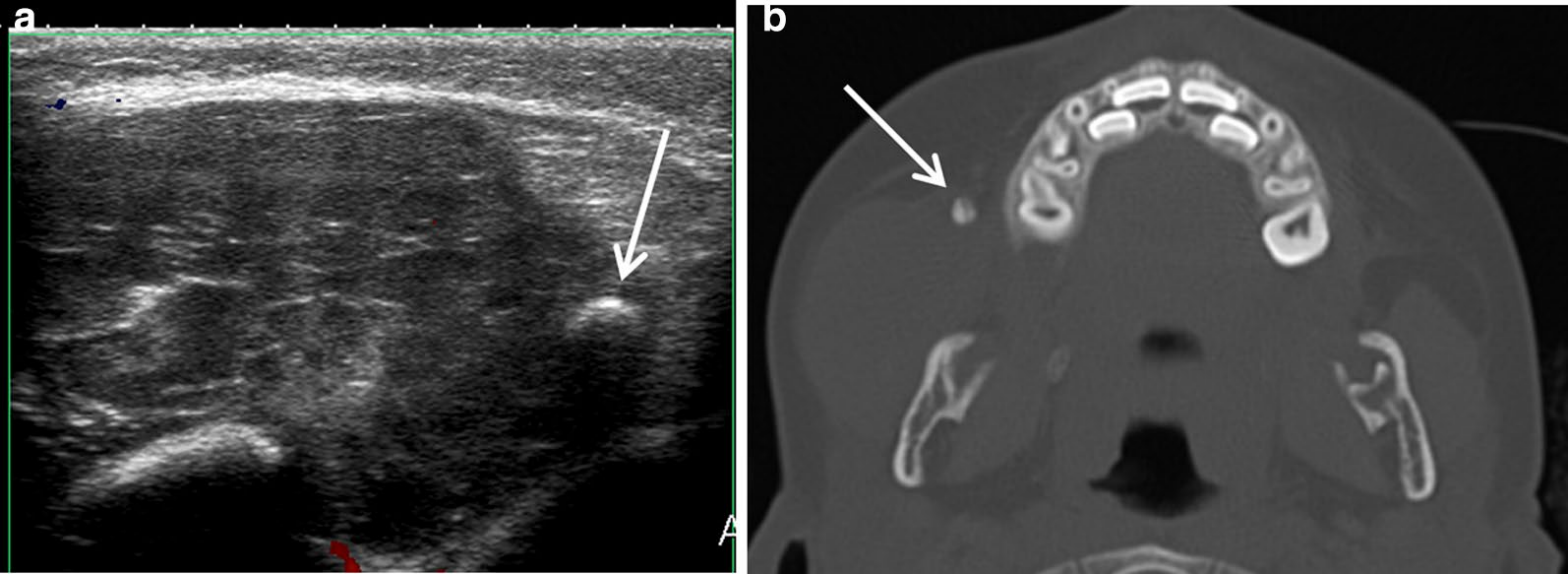
Venous malformation in the right masseter, with a typical phlebolith, on ultrasonography (**a**) and axial CT image (**b**)

Finally, close pathologies leading to a regional hyperemia and a swelling of the parotid gland can look clinically like a primitive parotid disorder. Examples are tumors (parapharyngeal lymphoma, masseter muscle rhabdomyosarcoma and mandibular sarcoma), congenital disorders (masseter venous malformation) and infections (mandibular osteomyelitis, temporomandibular arthritis, mastoiditis, subcutaneous fat cellulitis) (Figs. 23, 24) [40]. In such cases, MRI and CT are particularly helpful, in addition to ultrasonography, to explore the deep spaces and bones.

Conversely, lesions arising from the accessory parotid gland should not be confused with lesions of other cheek structures [14].

## Conclusion

In childhood, there are various disorders of the parotid gland, with specificities related to their nature; their clinical presentation, including the age of the patient; and their imaging aspects. Knowledge of this set of diagnostic elements is essential, allowing for appropriate management (medical treatment, surgery, fine needle biopsy).

## Summary of clinical and radiological features

### Parotitis

- Acute viral parotitis: often bilateral; painful swelling, infectious syndrome; homogeneous enlargement, hyperemia; inconstant involvement of submandibular and sublingual glands.
- Acute bacterial parotitis: likely unilateral; painful swelling, infectious syndrome, adjacent erythema; sometimes suppurated, abscessed.
- Cat scratch disease: chronic, intra- and/or extraparotid necrotic lymphadenitis; serology and/or PCR.
- Tuberculosis and mycobacterial infection: presenting like an acute parotitis or mimicking a tumor, necrotic, calcified lymphadenitis, skin fistula.
- Juvenile recurrent parotitis: uni- or bilateral; recurrent episodes of painful swelling; “panther dress” on imaging.

### Congenital anomalies

- Lymphatic malformation: soft, painless mass in the absence of complication; thin-walled macro- and/or microcysts; possible fluid–fluid level and trans-spatial extension.
- Venous malformation: bluish skin; soft, painless mass except in case of acute thrombosis; compressible mass or veins, phleboliths.
- First branchial cleft anomalies: congenital sinus or fistula (Pochet’s triangle or external auditory canal), parotid space cyst, pseudoabscess when infected.
- Aplasia: commonly unilateral, xerostomia and its complications when bilateral, possible associated malformation (Treacher-Collins).

### Tumors

#### Benign tumors

- Infantile hemangioma: a few weeks or months after birth; triphasic course; inconstant cutaneous infantile hemangioma; soft, painless mass; hypervascular tumor (Doppler: high-velocity and low-resistance arteries, MRI: flow voids, strong enhancement).
- Pleomorphic adenoma: after the age of 10 years; painless, slow-growing mass; well-defined tumor hypointense; capsule on *T*_2_-weighted image; high ADC.
- Neurofibroma: possible association with neurofibromatosis type 1; “target sign”: hypointense center and hyperintense periphery on *T*_2_-weighted image.

### Malignant tumors

- Mucoepidermoid carcinoma: asymptomatic mass; variable aspect on imaging: hyperintense area on T_2_-weighted image and low ADC in low-grade tumor (in most cases); inconstant lymphadenopathy (mainly in case of high-grade tumor).
- Rhabdomyosarcoma: young child and teenager; poorly defined mass, locally aggressive (possible bony destruction), with low ADC and diffuse and mild enhancement; lymphadenopathy.
- Sialoblastoma: fetus, newborn or infant; very rare; aggressive tumor with necrosis, hemorrhage and skin deformities.
- Lymphoma/leukemia: frequently bilateral; nodular masses (lymphoma); or diffuse infiltration (lymphoma/leukemia).

### Other disorders

- *Sialadenosis* late childhood and adolescence; eating disorders; bilateral, symmetrical enlargement with normal vascularization.
- *HIV infection* sometimes revealing the viral infection; bilateral, diffuse enlargement, multiple cysts and/or solid masses, regional lymphadenopathy.
- *Sarcoidosis* rare; painless, bilateral enlargement; homogeneous hypervascularization; possible involvement of lacrimal glands.
- *Sjogren’s disease* recurrent episodes of acute, tender parotid swelling (heterogeneous enlargement due to foci of mucoid impactions and ductal dilatations) and late atrophy and calcifications.

## Data Availability

All the original images are available from the corresponding author.

## Abbreviations

ADC: Apparent diffusion coefficient
CT: Computed tomography
DCE-MRI: Dynamic contrast-enhanced-magnetic resonance imaging
HIV: Human immunodeficiency virus
JRP: Juvenile recurrent parotitis
MRI: Magnetic resonance imaging
TBF: Tumor blood flow
TIC: Time intensity curve
Ve: Extracellular volume ratio
VM: Venous malformation

## References

[1] Sodhi KS, Bartlett M, Prabhu NK (2011). Role of high resolution ultrasound in parotid lesions in children. Int J Pediatr Otorhinolaryngol.

[2] García CJ, Flores PA, Arce JD, Chuaqui B, Schwartz DS (1998). Ultrasonography in the study of salivary gland lesions in children. Pediatr Radiol.

[3] Stern JS, Ginat DT, Nicholas JL, Ryan ME (2015). Imaging of pediatric head and neck masses. Otolaryngol Clin N Am.

[4] Shah GV (2002). MR imaging of salivary glands. Magn Reson Imaging Clin N Am.

[5] Freling N (2000). Imaging of salivary gland disease. Semin Roentgenol.

[6] Xu Z, Zheng S, Pan A, Cheng X, Gao M (2019). A multiparametric analysis based on DCE-MRI to improve the accuracy of parotid tumor discrimination. Eur J Nucl Med Mod Imaging.

[7] Yabuuchi H, Kamitani T, Sagiyama K (2020). Characterization of parotid gland tumors: added value of permeability MR imaging to DWI and DCE-MRI. Eur Radiol.

[8] Razek AAKA (2019). Multi-parametric MR imaging using pseudo-continuous arterial-spin labeling and diffusion-weighted MR imaging in differentiating subtypes of parotid tumors. Magn Reson Imaging.

[9] Yabuuchi H, Matsuo Y, Kamitani T (2008). Parotid gland tumors: can addition of diffusion-weighted MR imaging to dynamic contrast-enhanced MR imaging improve diagnostic accuracy in characterization?. Radiology.

[10] Abdel Razek AA, Samir S, Ashmalla GA (2017). Characterization of parotid tumors with dynamic susceptibility contrast perfusion-weighted magnetic resonance imaging and diffusion-weighted MR imaging. J Comput Assist Tomogr.

[11] Attyé A, Karkas A, Troprès I (2016). Parotid gland tumours: MR tractography to assess contact with the facial nerve. Eur Radiol.

[12] Carlson GW (2000). The salivary glands. Embryology, anatomy, and surgical applications. Surg Clin N Am.

[13] Som PM, Curtin HD (2003). Head and neck imaging.

[14] Currarino G, Votteler TP (2006). Lesions of the accessory parotid gland in children [published correction appears in Pediatr Radiol. Apr;36(4):376]. Pediatr Radiol.

[15] Francis CL, Larsen CG (2014). Pediatric sialadenitis. Otolaryngol Clin N Am.

[16] Agaimy A, Ito H, Zenk J (2017). Pediatric salivary gland tumors and tumor-like lesions. Pathologe.

[17] Hviid A, Rubin S, Mühlemann K (2008). Mumps. Lancet.

[18] UNICEF data—Pediatric care and treatment (2021)

[19] Dave SP, Pernas FG, Roy S (2007). The benign lymphoepithelial cyst and a classification system for lymphocytic parotid gland enlargement in the pediatric HIV population. Laryngoscope.

[20] Friedman E, Patino MO, Udayasankar UK (2018). Imaging of pediatric salivary gland. Neuroimaging Clin N Am.

[21] Saarinen RT, Kolho K-L, Pitkäranta A (2007). Cases presenting as parotid abscesses in children. Int J Pediatr Otorhinolaryngol.

[22] Melville DM, Jacobson JA, Downie, Biermann JS, Kim SM, Yablon CM (2015). Sonography of cat scratch disease. J Ultrasound Med.

[23] Inarejos Clemente EJ, Navallas M, Tolend M (2018). Imaging evaluation of pediatric parotid gland abnormalities. Radiographics.

[24] Boyd ZT, Goud AR, Lowe LH, Shao L (2009). Pediatric salivary gland imaging. Pediatr Radiol.

[25] Lowe LH, Stokes LS, Johnson JE (2001). Swelling at the angle of the mandible: imaging of the pediatric parotid gland and periparotid region. Radiographics.

[26] Gadodia A, Seith A, Sharma,  (2010). MRI and MR sialography of juvenile recurrent parotitis. Pediatr Radiol.

[27] Premnath KPB, Thomas J, Ray B, Jayakrishnan V (2016). Multimodality diagnostic features and treatment by sialography of juvenile recurrent parotitis: a case report. Int J Sci Study.

[28] SSVA Classification of Vascular Anomalies 2018 International Society for the Study of Vascular Anomalies. https://issva.org/classification. Accessed Jan 2019

[29] Bansal AG, Oudsema R, Masseaux JA, Rosenberg HK (2018). US of pediatric superficial masses of the head and neck. Radiographics.

[30] Turkington JR, Paterson A, Sweeney LE (2005). Neck masses in children. Br J Radiol.

[31] Dubois J, Alison M (2010). Vascular anomalies: what a radiologist needs to know. Pediatr Radiol.

[32] Meuwly JY, Lepori D, Theumann N (2005). Multimodality imaging evaluation of the pediatric neck: techniques and spectrum of findings. Radiographics.

[33] Maithani T, Pandey A, Dey D, Bhardwaj A, Singh VP (2014). First branchial cleft anomaly: clinical insight into its relevance in otolaryngology with pediatric considerations. Indian J Otolaryngol Head Neck Surg.

[34] Singh RP, Abdel-Galil K, Harbottle M, Telfer MR (2012). Parotid gland disease in childhood: diagnosis and indications for surgical intervention. Br J Oral Maxillofac Surg.

[35] Higley MJ, Walkiewicz TW, Miller JH, Curran JG, Towbin RB (2010). Aplasia of the parotid glands with accessory parotid tissue. Pediatr Radiol.

[36] Pham Dang N, Picard M, Mondié JM, Barthélémy I (2010). Complete congenital agenesis of all major salivary glands: a case report and review of the literature. Oral Surg Oral Med Oral Pathol Oral Radiol Endod.

[37] Bentz BG, Hughes CA, Lüdemann JP, Maddalozzo J (2000). Masses of the salivary gland region in children. Arch Otolaryngol Head Neck Surg.

[38] Morse E, Fujiwara RJT, Husain Z, Judson B, Mehra S (2018). Pediatric salivary cancer: epidemiology, treatment trends and association of treatment modality with survival. Otolaryngol Head Neck Surg.

[39] Baker SR, Malone B (1985). Salivary gland malignancy in children. Cancer.

[40] Koch BL, Myer CM (1999). Presentation and diagnosis of unilateral maxillary swelling in children. Am J Otolaryngol.

[41] Menapace D, Mitkov M, Towbin R, Hogeling M (2016). The changing face of complicated infantile hemangioma treatment. Pediatr Radiol.

[42] Rotter A, Samorano LP, Rivitti-Machado MC, Oliveira ZNP, Gontijo B (2018). PHACE syndrome: clinical manifestations, diagnostic criteria, and management. An Bras Dermatol.

[43] Williams MF, Ellis GL, Auclair PL, Gnepp DR (1991). Salivary gland neoplasms. Surgical pathology of the salivary glands.

[44] Hornigold R, Morgan PR, Pearce A (2005). Congenital sialolipoma of the parotid gland first reported case and review of the literature. Int J Pediatr Otorhinolaryngol.

[45] Bollig C, Moon S, Sujoy V, Younis R (2014). Castleman disease of the parotid in childhood: a case report. Am J Otolaryngol.

[46] Sultan I, Rodriguez-Galindo C, Al-Sharabati S, Guzzo M, Casanova M, Ferrari A (2011). Salivary gland carcinomas in children and adolescents: a population-based study, with comparison to adult cases. Head Neck.

[47] Yoshida EJ, Garcia J, Eisle DW (2014). Salivary gland malignancies in children. Int J Pediatr Otolaryngol.

[48] Krolls SO, Trodahl JN, Boyers RC (1972). Salivary gland lesions in children. A survey of 430 cases. Cancer.

[49] Ellies M, Laskawi R (2010). Diseases of the salivary glands in infants and adolescents. Head Face Med.

[50] Christe A, Waldherr C, Hallett R, Zbaeren P, Thoeny H (2011). MR imaging of parotid tumors: typical lesion characteristics in MR imaging improve discrimination between benign and malignant disease. AJNR Am J Neuroradiol.

[51] Kashiwagi N, Dote K, Kawano K (2012). MRI findings of mucoepidermoid carcinoma of the parotid gland: correlation with pathological features. Br J Radiol.

[52] Abdel Razek AA, Gabalia G, Elhawarey G (2009). Characterization of pediatric head and neck masses with diffusion-weighted MR imaging. Eur Radiol.

[53] Ord RA, Carlson ER (2016). Pediatric salivary gland malignancies. Oral Maxillofac Surg Clin N Am.

[54] Thariat J, Vedrine P-O, Orbach D (2011). Salivary gland tumors in children. Bull Cancer.

[55] Rebours C, Couloigner V, Galmiche L (2017). Pediatric French Rare Tumor Group. Pediatric salivary gland carcinomas: diagnostic and therapeutic management. Laryngoscope.

[56] Som PM, Brandwein M, Silvers AR, Rothschild MA (1997). Sialoblastoma (embryoma): MR findings of a rare pediatric salivary gland tumor. AJNR Am J Neuroradiol.

[57] Saha A, Dandekar S, Milla S, Roman E, Bhatla T (2014). Bilateral parotid gland enlargement and palpable nephromegaly in infant acute lymphoblastic leukemia: case report and review of the literature. J Pediatr Hematol Oncol.

[58] Mesa JR, Espinosa E, Losada R, Hernandez C, Martinez G, Hernandez P (1999). Parotid and Central nervous system relapse during complete hematologic remission in acute promyelocytic leukemia. Haematologica.

[59] Millman B, Pellitteri PK (1995). Thyroid carcinoma in children and adolescents. Arch Otolaryngol Head Neck Surg.

[60] Gritzmann N, Rettenbacher T, Hollerweger A, Macheiner P, Hübner E (2003). Sonography of the salivary gland. Eur Radiol.

[61] Iqbal Y, Al-Shaalan M, Al-Alola S (2004). Langerhans cell histiocytosis presenting as a painless bilateral swelling of the parotid glands. J Pediatr Hematol Oncol.

[62] Koyama T, Ueda H, Togashi K (2004). Radiologic manifestations of sarcoidosis in various organs. Radiographics.

[63] Cimaz R, Casadei A, Rose C (2003). Primary Sjögren syndrome in the paediatric age: a multicentre survey. Eur J Pediatr.

[64] Nikitakis NG, Rivera H, Lariccia C, Papadimitriou JC, Sauk JJ (2003). Primary Sjögren syndrome in childhood: report of a case and review of the literature. Oral Surg Oral Med Oral Pathol Oral Radiol Endod.

[65] Tonami H, Ogawa Y, Matoba M (1998). MR sialography in patients with Sjögren syndrome. AJNR Am J Neuroradiol.

[66] Prabhu SP, Tran B (2008). Pneumoparotitis. Pediatr Radiol.

[67] Gazia F, Freni F, Galletti C (2020). Pneumoparotid and pneumoparotitis: a literary review. Int J Environ Res Public Health.

