# Correction: Imaging of parotid anomalies in infants and children

**DOI:** 10.1186/s13244-022-01231-6

**Published:** 2022-05-06

**Authors:** François Chalard, Anne-Laure Hermann, Monique Elmaleh-Bergès, Hubert Ducou le Pointe

**Affiliations:** 1 Department of Pediatric Radiology, Hôpital Armand Trousseau, 26, Avenue du Dr. Arnold Netter, 75012 Paris, France; 2 Pediatric Radiology, Hôpital Robert Debré, Paris, France

## Correction: Insights into Imaging (2022) 13:27 10.1186/s13244-022-01166-y

The original article [1] mistakenly omitted arrows in some figures which have all since been restored.
